# The prevalence and multilevel analysis of minimum dietary diversity intake and its determinants among 6–23 months old infants in The Gambia: further analysis of the Gambian demographic and health survey data

**DOI:** 10.1186/s41043-023-00442-x

**Published:** 2023-09-13

**Authors:** Bewuketu Terefe, Mahlet Moges Jembere, Nega Tezera Assimamaw

**Affiliations:** 1 Department of Community Health Nursing, School of Nursing, College of Medicine and Health Sciences, University of Gondar, Po. Box: 196, Gondar, Amhara Ethiopia; 2 Department of Emergency and Critical Care Nursing, School of Nursing, College of Medicine and Health Sciences, University of Gondar, Gondar, Amhara Ethiopia; 3 Department of Pediatrics and Child Health Nursing, School of Nursing, College of Medicine and Health Sciences, University of Gondar, Gondar, Amhara Ethiopia

**Keywords:** Prevalence, Determinants, Multilevel analysis, Minimum dietary diversity, 6–23 months old infants, The Gambia, GDHS

## Abstract

**Background:**

Poor infant and young child feeding (IYCF) practices are a significant issue both globally and in developing nations, and they have a significant role in undernutrition, healthy growth, and development, particularly in the first 2 years of life. Improving children's general health and wellbeing requires recognizing and decreasing preventable drivers of malnutrition. Hence, this study aimed to assess the prevalence and possible determinants of minimum dietary diversity among 6–23 months old babies in the Gambia.

**Methods:**

Data from the 2019–2020 Gambia demographic and health survey were used. The study included a total of 2100 weighted 6–23 months old children. To identify characteristics significantly linked with minimum dietary diversity among infants, a multilevel fixed-effect analysis approach was used. After adjusting other confounding variables, variables with a *p* value of 0.25 were incorporated into a multivariable multilevel regression analysis to determine associated variables. An adjusted odds ratio with a 95% confidence interval was then applied.

**Results:**

Only 22.22% (95% CI 18.55, 21.99) of infants had received the minimum dietary diversity. Mothers, who had mass media exposure (aOR = 2.71, CI = (1.02, 6.21), wealthier (aOR = 1.70, CI = 1.02, 2.85), child age of (aOR = 4.14, CI = 2.98, 5.76), and (aOR = 4.97, CI = 3.54, 6.98), have shown a positive statistical association with the outcome variable, respectively. Regarding regions mothers who came from Kanifing (aOR = 0.49, CI = 0.25, 0.94), Janjanbureh (aOR = 0.38, CI = 0.18, 0.82), and Basse (aOR = 0.51, CI = 0.26, 0.99) had showed less likelihood odds to provide the minimum dietary diversity (MDD) for their babies compared to Banjul local government area, respectively.

**Conclusion:**

The World Health Organization dietary evaluation tool suggests that the MDD value be extremely low, even though it might be slightly higher than the numbers for some nations. The country may need to take drastic measures to tackle child malnutrition.

## Introduction

Nutritional status and micronutrient intake in children are currently predicted by dietary diversity (DD). DD is a straightforward indicator that is frequently used as a stand-in for dietary quality, sufficiency in micronutrients, and food accessibility [1, 2]. The World Health Organization (WHO) defines DD as eating foods from the key nutritionally important diet kinds while keeping a balance between meals derived from plant and animal sources [3, 4]. For higher dietary quality and to meet daily energy and nutrient needs, minimum dietary diversity (MDD) is the consumption of five or more food groups from the eight recommended food groups, including breast milk, grains, roots, and tubers, legumes, and nuts; dairy products (infant formula, milk, yoghurt, and cheese); flesh foods (meat, fish, poultry, and liver/organ meats); eggs; vitamin A-rich fruits and vegetables; and other fruits and vegetables [3–5].

Children mainly under five are suffering tremendously by malnutrition in all regions of the globe; however, its negative consequences are deeply rooted in Africa [6, 7]. All of those African nations are severely hit by this public challenges socially, economically, and politically [8]. All nations, stakeholders, intersectoral organizations, governmental and non-governmental bodies should put their positive impact to reduce the severity of malnutrition around the globe. Given the scale and severity of the problem, various health care organizations have made significant contributions by regularly creating awareness, assigning and training health professionals, and developing materials and treatment guidelines [9]. In 2002, the WHO, in collaboration with the UNICEF, launched a global feeding strategy called infant and child feeding (IYCF) to reverse the low trends of child feeding practices [9].

The importance of halting undernutrition from getting children is because of, it affects about 149.2 million children of under 5 years of age were stunted and 45 million are exposed to wasted globally in 2020, with 75% of such children living in the African and Southeast Asia Regions [10, 11]. The 2019 regional analysis report indicated that 57.5 million, 11.8 million and 3.0 million under five children were exposed to stunting, wasting and severely wasting [7]. To maintain proper physical growth and metal development, young children and infants should take a minimum of five from the total balanced foods of namely, grains, roots, and tubers; legumes and nuts; dairy products; flesh foods (meat, fish, poultry, and organ meats); eggs; vitamin-A-rich fruits and vegetables; and other fruits and vegetables according to WHO recommendation [12–14], nevertheless, the recommendation did not accomplish as it stated by many developing nations [15, 16]. From the perspective of these studies, it is possible to conclude that household food insecurity is a factor for inadequate dietary consumption in Africa [17, 18]

According to the facts of several studies, inadequate dietary intake is one of the major causes of life-threatening illnesses by leading children to stunting, underweight and wasting [12]. Malnutrition can have a long-lasting impact on children's health and well-being. It can lead to chronic diseases, such as heart disease, stroke, and diabetes, later in life. It can also reduce children's chances of success in school and in life [10]. Children don not only endanger the lives of children, but also challenge the survival of their families and nations by predisposing them from other diarrheal diseases and infections, as well as inadequate participation, performance in their communities and schools also have great chance to happened [19–21]. Studies depicted that place of residence, age of the child, maternal education, birth order, wealth index and number of children in the household, high burden of disease and malnutrition practices, home gardening and media exposure were some of the factors which associated with MDD [22–25]

Nutrition is one of the major issues the world is implementing on by 2030 to eradicate hunger [26]. However, most parents and care givers in the developing countries find it difficult to provide adequate and diversified nutrition for their children [27]. This is likely to be a major challenge for the world in its 2030 to eradicate hunger, and various remedial measures are needed [15, 16].

According to the 2018 UNICEF MICS report, malnutrition is keeping going as a big deal of Gambian children. As a result of poor health services, limited knowledge of parents, most children did not feed vitamins, proteins and minerals reduction [28]. This finding is supported by the 2021 Global Nutrition Report, which noted that the Gambia has not made much progress in reducing stunting among young children and noncommunicable diseases linked to diet [29]. This role of this study is to prove information to the harmed of the inadequate dietary nutrition in Gambia, particularly those aged 6–23 months for the implementation to reduce undernutrition among infants and young children in Gambia.

## Methods and materials

### Study design and setting

The Gambia Demographic Health Survey (GDHS), a secondary, sizable community-based cross-sectional survey that was carried out in the Gambia from November 21, 2019, to March 30, 2020, served as the foundation for this investigation. The Gambia Bureau of Statistics (GBoS) in collaboration with the Ministry of Health and Social Welfare performed the 2019–2020 Gambia Demographic and Health Survey (GDHS 2019–2020) between October 2019 and February 2020. The Gambia Demographic and Health Survey (GDHS 2019–2020) is the second DHS survey that was conducted in the Gambia in cooperation with the global Demographic and Health Survey Program. The Gambia is a country in West Africa. The Republic of Senegal forms its northern, southern, and eastern borders, while the Atlantic Ocean forms its western boundary. The nation experiences two distinct seasons, the rainy season (June to October) and the dry season (November to May) [30].

### Source population and sampling technique

Stratified, two-stage cluster sampling was used for the survey. Enumeration areas (EAs) inside each sampling stratum were initially chosen with a probability proportional to their size. The households were carefully sampled in the second step. Each of the chosen clusters or EAs were chosen with a probability proportional to their size inside each sampling stratum in the first step. A total of 281 EAs were chosen. A set number of 25 households were systematically chosen from the resulting lists of households as the sampling frame, yielding a total sample size of 7025 selected households. Results from this sample are reflective of those found across the nation, in urban, rural, and local government areas [30]. Mothers who had children with 6–23 months old 24 h before to the survey made up the source population. In order to correct for unequal sample distributions during data collection, the estimation process applied the weightings for the children's sample. As a result, 2100 weighted samples of children at age of 6–23 months were used in the study. The detail can be accessed from the GDHS report (https://dhsprogram.com/pubs/pdf/FR369/FR369.pdf).

### Study tools and measurement

#### Dependent variable

The 24-h recall of food groups from the GDHS survey was used to build the dietary diversity indicator that was used in the study [30]. Guidelines from WHO and UNICEF define the MDD score for children aged 6–23 months as the percentage of children who ate anything from at least five of the eight food groups within a 24-h period. 1-grains, roots and tubers, 2-legumes and nuts, 3-dairy products, 4-meat, fish, poultry, and liver/organ meats, 5-eggs, 6-vitamin A-rich fruits and vegetables, 7-other fruits and vegetables, and 8-breast milk are the eight food groups [4, 30, 31]. In this study, the MDD score of the children was divided into two categories. Children between the ages of 6 and 23 months who ate at least five different food types in the 24 h before an interview are deemed to have complied with the MDD requirements and their MDD score is classified as having sufficient minimal dietary diversity. Additionally, kids between the ages of 6 and 23 months who ingested fewer than five food categories in the 24 h preceding an interview were deemed to have fallen short of the MDD standards, with their MDD score being classified as having inadequate minimal dietary diversity [31].

#### Independent variable

The literature review of factors linked to children's DD served as a guide for the study's design and creation of the conceptual framework. As result, both individual and community level factors were assessed to determine the highest potential factors that are linked with MDD in the Gambia. Personal-related factors such as maternal age, educational attainment, sex of the household head, wealth index, marital status, child age, ANC, PNC after 02 months, place of delivery, family size, twins, under five children, size of the child, media exposure (watching to TV, listening to radio and reading to magazine/newspapers), breast feeding status, order of birth, occupation, whereas, in the community level factors such as region, residence, community level poverty, community level education, community media exposure, community, and ANC utilization were included in the model.

### Data management and analysis process

We did a secondary analysis of the GDHS 2019–2020 using the Kids Records (KR) dataset. STATA version 17 and Microsoft Excel version 19 were used for data purification. Calculations and descriptive statistics, such as frequency and percentages of different variables, were provided using texts, tables, and graphs. A multilevel mixed effect logistic regression model was applied to each independent variable, and only variables with *p* values lower than 0.25 were considered. The adjusted odds ratio was used to calculate the degree of connection between the dependent and independent variables, and variables with a *p* value of < 0.05 were regarded as statistically significant.

In the GDHS data, the infant is contained within a cluster, and neonates from the same cluster showed more resemblance than neonates from other clusters. As a result, the common regression model's assumptions regarding observation independence and equal variance across clusters are broken. This suggests that in order to take into consideration between-cluster effects, a complex model must be used. A multilevel random intercept logistic regression model was created to ascertain the association between individual-level and community-level characteristics and the chance that a newborn will not receive postnatal care within 2 days of birth.

There was a total of four models created. Without any explanatory variables, the first model—also referred to as an empty or null model—was fitted. The disparities across communities were lessened by using this tactic. Understanding community variances requires knowledge of the null model. As a starting point, we evaluated how much the observed discrepancies in mothers' behavior regarding MDD may be attributed to societal influences. Additionally, this model served as evidence in favor of the adoption of a multi-level statistical framework and a yardstick for determining whether to utilize multi-level or traditional logistic regression. It was evaluated using the proportional change of variance (PCV), the log-likelihood ratio test (LLR), the median odds ratio (MOR), the intraclass correlation coefficient (ICC), and the AIC. Only individual-level characteristics were included in the second model. Only community-level features were present in the third. In contrast, the final (fourth) model included both elements at the individual and community levels. Additionally, when comparing models using model deviance, the model with the lowest deviation was picked for reporting and result interpretation.

In order to compare the layered models, deviation (2log likelihood) was utilized. The intracellular correlation coefficient (ICC) and log-likelihood were used to calculate the variation between clusters. The ICC displays the degree of variability among infants who do not receive MDD before 24 h of the interview. A multilevel binary logistic regression analysis was conducted to identify the individual and societal variables that influence 6–23-month-old infants' MDD before 24 h of the interview.

$${\text{Log}}\left( {\pi ij \div 1 - \pi ij} \right) = \beta o + \beta 1xij + \beta 2xij + \cdots uj + eij$$

where *πij* is the likelihood that no iron will be consumed, and ij is the likelihood that it will. When none of the explanatory factors are present, the influence on MDD is represented by the intercept, or *β*0. The variables at the individual and community levels for the *i*th person in group *j* are *βxij*, respectively. Also, because the *β*'s are fixed coefficients, a rise in *X* can result in an increase in the risk of ingesting iron by an additional *ß* unit. The UK demonstrates the *j*th community's random effect—the influence of the community on the mother's decision to intake MDD. Assuming that each cluster has a unique intercept (*β*0) and fixed coefficient (*β*), the clustered nature of the data as well as between and between community variances were taken into consideration.

The likelihood test was used to compare the models, and model 4 was found to have the greatest value and be the best fit. All variables had VIF values less than 10, and the mean VIF value of the final model was 1.50, which was utilized to identify multicollinearity. Crude odds ratio (COR) and adjusted odds ratio were used to measure the relationship between the dependent and independent variables (AOR). For the final model, factors having a *p* value of less than 0.25 in COR have been chosen as contenders. Adjusted odds ratios and 95% confidence intervals with a *p* value of 0.05 were used to determine the strength of associations between dependent and independent factors. The median odds ratio (MOR), which is defined as the median value of the odds ratio between the area at the lowest risk and the highest risk when randomly choosing two clusters, was used to assess the measure of variance. MOR = e0.95√VA or, MOR = exp. [√(2 × VA) × 0.6745], where VA is the area level variance [32, 33]. The Proportional Change in Variance (PCV) reveals the variation in MDD intake among children 6–23 months explained by factors. The PCV is calculated as = $\frac{{{\text{Vnull}} - {\text{VA}}}}{{{\text{Vnull}}}}$*100, where Vnull is the initial model's variance, and VA is the model's variance with additional terms. Also, the intraclass correlation coefficient (ICC), a measurement of the variation in bottle feeding between clusters, is computed as; ICC = VA ÷ VA + 3.29 ∗ 100%, where VA = area/cluster level variance [32, 33].

### Operational definitions

*Community women education* This represents the overall worth of women's educational attainment as determined by the median distribution of educational attainment in the neighborhood. The ratio of women in the community with at least a secondary education was considered low if it fell below the median (0–8.33%), and high if it rose above the median (8.34–100%).

*Community media exposure* This variable was derived from individual responses to radio or television media exposure. It was defined as low if the proportion of women exposed to media in the community was 0–66% and high if the proportion was 66.67–100%.

*Community ANC utilization rate* This variable is also produced from the various ANC utilization individual values. If the proportion of women who attended at least three ANC visits in the community ranged from 0 to 82%, it was considered low. 0.83 was the median value.

*Community poverty* The same process is used to get this variable from each household's wealth index. It was deemed high if the proportion of women in a community's two lowest wealth quintiles was between 56 and 100%, and low if it was between 0 and 55%.

## Results

### Sociodemographic characteristics of the study participants

To assess the MDD intake a total of 2100 weighted mothers were included in this study. Slightly more than half 1126 (53.63%), of them and nearly half 940 (44.77%) of the study participants were with an estimated age range of 25–34 years old and did not enroll in formal education, respectively; however almost nearly all of them 1969 (93.79%) were married and 472 (22.50%) were came from poorest household, respectively. Regarding ANC visits and types of place of delivery of the house about 1702 (81.03%), and 1846 (87.93%), had shown at least three visits and institutional delivery, respectively. Majority 1733 (82.55%) and slightly higher half 1172 (55.86%) of the study participants have shown currently working status and baby PNC checkup after 02 months. Moreover, about 1212 (57.73%) mothers came from large family members (more than 10), and large portion of household heads are male 1798 (85.61%). Regarding children’s profile about 922 (43.92%), 1094 (52.11%), and 916 (43.64%) of them were found in at large birth weight, male and fourth and more order of birth, respectively. About 782 (37.23%), and 2058 (98.02%) babies are found from 11 to 17 months old and single birth, respectively (Table 1).

**Table 1:** Individual level factors of sociodemographic characteristics of study participants on MDD intake among mothers and 6–23 months old children in Gambia, GDHS 2019/20, (*n* = 2100)

Variables	MDD intake		Total, *n* (%)
	No, *n* (%)	Yes, *n* (%)	
Maternal age in years			
15–24	451 (83.63)	88 (16.37)	539 (25.66)
25–34	889 (78.97)	237 (21.03)	1126 (53.63)
35–49	335 (77.13)	100 (22.87)	435 (20.72)
Maternal education			
No formal education	786 (83.59)	154 (16.41)	940 (44.77)
Primary	321 (77.11)	95 (22.89)	416 (19.84)
Secondary	512 (77.85)	145 (22.15)	657 (31.30)
Higher	57 (65.85)	29 (34.15)	86 (4.09)
Marital status			
Not married	104 (79.46)	27 (20.54)	131 (6.21)
Married	1571 (79.80)	398 (20.20)	1969 (93.79)
Wealth index			
Poorest	403 (85.33)	69 (14.67)	472 (22.50)
Poorer	368 (83.94)	70 (16.06)	438 (20.88)
Middle	335 (74.87)	112 (25.13)	447 (21.29)
Richer	314 (77.07)	94 (22.93)	408 (19.41)
Richest	255 (76.36)	79 (23.64)	334 (15.92)
Currently working			
No	870 (84.99)	154 (15.01)	1023 (48.73)
Yes	806 (74.84)	271 (25.16)	107 (51.27)
ANC follow up			
Less than four	309 (77.57)	89 (22.43)	398 (18.97)
Four and above	1366 (80.30)	335 (19.70)	1702 (81.03)
Place of delivery			
Home	202 (79.78)	51 (20.22)	253 (12.07)
Health facility	1473 (79.78)	373 (20.22)	1846 (87.93)
Currently breastfeeding status			
No	299 (81.46)	68 (18.54)	367 (17.45)
Yes	1376 (79.43)	357 (20.57)	1733 (82.55)
Postnatal checkup after 02 months			
No	727 (78.45)	200 (21.55)	926 (44.14)
Yes	948 (80.85)	224 (19.15)	1172 (55.86)
Family size			
2–6	318 (81.58)	72 (18.42)	390 (18.54)
7–10	390 (78.19)	109 (21.81)	498 (23.73)
More than 10	968 (79.86)	244 (20.14)	1212 (57.73)
Sex of the household head			
Male	1440 (80.11)	358 (19.89)	1798 (85.61)
Female	235 (77.86)	67 (22.14)	302 (14.39)
Reading to magazine/newspaper			
No	1646 (80.33)	403 (19.67)	2049 (97.61)
Yes	29 (57.46)	22 (42.54)	51 (2.39)
Watching to television			
No	834 (80.36)	204 (19.64)	1038 (49.46)
Yes	841 (79.22)	221 (20.78)	1062 (50.54)
Listing to radio			
No	1035 (80.52)	250 (19.48)	1285 (61.21)
Yes	641 (78.62)	174 (21.38)	815 (38.79)
Child birth weight			
Large	730 (79.16)	192 (20.84)	922 (43.92)
Average	675 (80.53)	163 (19.47)	838 (39.93)
Small	270 (79.63)	69 (20.37)	339 (16.15)
Sex of the child			
Male	867 (79.24)	227 (20.76)	1094 (52.11)
Female	808 (80.37)	197 (19.63)	1006 (47.89)
Birth order			
First	346 (81.01)	81 (18.99)	428 (20.36)
2nd and 3rd	602 (79.59)	154 (20.41)	756 (35.99)
4th and above	727 (79.36)	189 (20.64)	916 (43.64)
Twin status			
No	1636 (79.51)	422 (20.49)	2058 (98.02)
Yes	39 (93.47)	3 (6.53)	42 (1.98)
Number of under five children			
0–2	741 (78.06)	209 (21.94)	950 (45.24)
More than 02	934 (81.21)	216 (18.79)	1150 (54.76)
Child age in months			
6–11	669 (92.20)	57 (7.80)	726 (34.58)
12–17	584 (74.66)	198 (25.34)	782 (37.23)
18–23	422 (71.32)	170 (28.68)	592 (28.20)

### Community level factors characteristics of the study participants

As it illustrated in the following table regarding community literacy, media exposure, ANC utilization, regions, residence, and poverty proportions nearly half of them 1127 (53.67%) had low ANC follow-ups, low community poverty level, 1381 (65.74%) media exposure 1138 (54.17%). Large portion of the study participants came from Brikama, and urban areas 866 (41.23%), and 1378 (65.64%), respectively (Table 2)

## Highlights of random effect analysis

Due to the hierarchical nature of the DHS data, we assessed the clustering effect. The null model's ICC in the random-effects investigation had a high value. This shows that the variance within clusters accounted for about 12.04% of the variety in MDD provision for babies, leaving individual variation to explain for the remaining 87.96%. The empty model's higher MOR value revealed a wide range in MDD provision between clusters. The MOR value of the empty model showed that if we observed two children from two different clusters, one would be more likely to use MDD provision than the other: a child in the cluster with high MDD utilization had a 1.90 times higher likelihood of using MDD provision than a child in the cluster with lower MDD intake. The model with the lowest deviation—in this case, Model III with a deviance of 2001.14—was chosen as the best-fit model since it was the smallest value. Deviation was also utilized to assess model fitness. Model III (Table 2) is the one to use in the ideal situation (Table 2).

**Table 2:** Community level factors characteristics of study participants on MDD intake among mothers and 6–23 months old children in Gambia, GDHS 2019/20, (*n* = 2100)

Variables	MDD intake		Total, *n* (%)
	No, *n* (%)	Yes, *n* (%)	
Community ANC utilization			
Low	878 (77.89)	249 (22.11)	1127 (53.67)
High	797 (81.98)	176 (18.02)	973 (46.33)
Community poverty			
High	611 (84.89)	109 (15.11)	719 (34.26)
Low	1065 (77.12)	316 (22.88)	1381 (65.74)
Community media exposure			
Low	790 (82.15)	172 (17.85)	962 (45.83)
High	885 (77.78)	253 (22.22)	1138 (54.17)
Community women education			
Low	612 (83.43)	122 (16.57)	734 (34.92)
High	1063 (77.83)	303 (22.17)	1366 (65.08)
Types of place of residence			
Urban	1065 (77.30)	313 (22.70)	1378 (65.64)
Rural	610 (84.52)	112 (15.48)	722 (34.36)
Region			
Banjul	13 (71.35)	5 (28.65)	18 (0.86)
Kanifing	297 (84.26)	55 (15.74)	352 (16.77)
Brikama	639 (73.84)	227 (26.16)	866 (41.23)
Mansakonko	77 (83.33)	15 (16.67)	92 (4.39)
Kerewan	211 (84.67)	38 (15.33)	249 (11.87)
Kuntaur	107 (80.19)	27 (19.81)	134 (6.37)
Janjanbureh	108 (86.58)	17 (13.42)	125 (5.96)
Base	223 (84.60)	41 (15.40)	264 (12.56)

### Determinants associated with MDD among 6–23-month-old children in the Gambia

This study declared some determinants including child age, wealth index, mass media exposure, and region were statistically significant in the multilevel mixed effect of logistic regression model with the outcome variable. It was evident that those mothers who have mass media exposure of reading to magazines/newspapers had revealed higher odds to provide the MDD supply for their children compared to their counterparts (aOR = 2.71, CI = (1.02, 6.21). Similarly, it was also revealed that those who have classified under richer wealth index, had higher odds to feed the MDD for their kids compared to with poorest mothers (aOR = 1.70, CI = 1.02, 2.85). Children whose age found from 12–17 and 18–23 months old have a higher odd of (aOR = 4.14, CI = 2.98, 5.76), and (aOR = 4.97, CI = 3.54, 6.98) times to receive the MDD compared to with 6–11 months old children, respectively. Conversely, regarding regions mother who came from Kanifing (aOR = 0.49, CI = 0.25, 0.94), Janjanbureh (aOR = 0.38, CI = 0.18, 0.82), and Basse (aOR = 0.51, CI = 0.26, 0.99) had showed less likelihood odds to provide the MDD for their babies compared to their compared to Banjul local government area, respectively (Table 3).

**Table 3:** Individual and community-level factors associated with MDD among 6–23 months old children in Gambia, GDHS 2019/20, (*n* = 2100)

Independent variables	Null model	Model I	Model II	Model III
		AOR (95% CI)	AOR (95% CI)	AOR (95% CI)
Wealth index				
Poorest		Ref.		Ref.
Poorer		0.93 (0.65, 1.34)		0.96 (0.66, 1.39)
Middle		1.54 (1.04, 2.21)		1.53 (0.99, 2.35)
Richer		1.70 (1.11, 2.59)		1.70 (1.02, 2.85) *
Richest		1.19 (0.72, 1.99)		1.19 (0.65, 2.19)
Educational status				
No formal education		Ref.		Ref.
Primary		1.20 (0.88, 1.63)		1.18 (0.87, 1.60)
Secondary		1.19 (0.89, 1.60)		1.17 (0.87, 1.57)
Higher		1.77 (0.88, 3.58)		1.74 (0.86, 3.49)
Watching to television				
No		Ref.		Ref.
Yes		0.96 (0.72, 1.27)		0.96 (0.72, 1.27)
Listening to radio				
No		Ref.		Ref.
Yes		1.15 (0.90, 1.46)		1.16 (0.91, 1.47)
Reading to magazine/newspaper				
No		Ref.		Ref.
Yes		2.71 (1.09, 6.73)		2.51 (1.02, 6.21) *
ANC follow-up				
At least three times		Ref.		Ref.
More than three times		0.83 (0.62, 1.11)		0.86 (0.63, 1.17)
Child age in month				
6–11		Ref.		Ref.
12–17		4.14 (2.98, 5.76)		4.14 (2.98, 5.76) *
18–23		4.99 (3.54, 7.01)		4.97 (3.54, 6.98) *
Community ANC utilization				
Low			Ref.	Ref.
High			0.85 (0.65, 1.12)	0.92 (0.69, 1.23)
Types of place of residence				
Urban			Ref.	Ref.
Rural			0.90 (0.61, 1.34)	1.18 (0.75, 1.85)
Regions				
Banjul			Ref.	Ref.
Kanifing			0.44 (0.23, 0.83)	0.49 (0.25, 0.94) *
Brikama			0.80 (0.45, 1.41)	0.86 (0.48, 1.56)
Mansakonko			0.51 (0.24, 1.06)	0.54 (0.25, 1.16)
Kerewan			0.46 (0.24, 0.91)	0.51 (0.25, 1.03)
Kuntaur			0.66 (0.33, 1.34)	0.82 (0.39, 1.69)
Janjanbureh			0.34 (0.16, 0.71)	0.38 (0.18, 0.82) *
Base			0.46 (0.24, 0.87)	0.51 (0.26, 0.99) *
Random parameters and model comparison				
Community-level variance	0.45 (0.25, 0.81)	0.39 (0.21, 0.75)	0.33 (0.17, 0.67)	0.31 (0.15, 0.66)
ICC (%)	12.04	10.61	9.24	8.65
MOR (CI, 95%)	1.90 (1.54, 2.25)	1.82 (1.46, 2.17)	1.74 (1.41, 2.07)	1.70 (1.36, 2.05)
PCV (%)	Reference	13.33	26.67	31.11
Log-likelihood (LLR)	− 1081.65	− 1009.21	− 1069.29	− 1000.57
DIC (-2LLR)	2163.30	2018.42	2138.58	2001.14

Ref. and * indicates reference variables and significance level at *p* value < 0.05

## Discussion

Using the most recent nationally representative data from the National Centre for demography and Health Statistics, this study examined mothers of 6- to 23-month-old children for minimum dietary diversity feeding in the Gambia. We found statistically significant relationships between local government areas, household wealth index, age of the child, and mass media exposure for reading newspapers or books with minimum dietary diversity feeding among mothers of 6–23-month-old children in the Gambia in multiple mixed effects multilevel logistic regressions or the final model of analysis that adjust for confounders. Based on this, just 20.22% (95% CI 18.55–21.99) of children aged 6–23 months in the Gambia had access to sufficient MDD, or around one in five children only. This is a worrying indication that the problem still poses a serious nutritional issue that the Gambia has to address by developing comprehensive nutritional strategy. This finding is nearly in line with studies conducted in Rwanda [34] and India [21]; however, this figure has shown a less MDD coverage compared to literatures done in Ethiopia, Bangladesh [35], and Nepal [36]. With contrast to studies conducted in Ethiopia [37], and 33 SSA recent study [38], this study has declared higher MDD supply for children, respectively.

According to the country's profile, the possible gap is caused by poor food production, consumption, living standard of the people, countries commitment to their policy, sociocultural and attitudes as well as maternal and child health services coverage, and people's ability to purchase a range of food items in Gambia. The recent rise in the cost of consumable goods and a lack of knowledge about how to make complementary foods may be factors in the lack of dietary diversity among Gambian youngsters.

The results of this study suggest that children between the ages of 6 and 23 months require a diet that is a little more varied than is typical for their age. When compared to a kid aged 6–11 months, a child between the ages of 12 and 17 and 18 and 23 months had around a four to five times greater likelihood of receiving the minimum amount of diverse food. We can support this with solid evidences we have in hand. Several studies done in Ethiopia [37, 39], Indonesia [40], Bangladesh [40], and Nepal [36] have declared similar findings with this study at hand. An evidence from the UNICEF found that whereas nearly half of the young children aged 18–23 months received four or more food categories each day, less than a quarter of infants aged 6–11 months did [41]. There is a significant age gap between the children in this. This can be a result of mothers' attitudes about IYCF. According to research on Gambia's eating habits and beliefs, many mothers do not consider starting supplemental feeding for their children at a young age [42]. Mothers' socioeconomic position, culture, and customs have been observed to affect the early introduction of supplemental foods in the Gambia and Nepal [42, 43]; furthermore, the study highlighted that this could be difficult without the help of their friends, families, and community. Therefore, it is vital to implement national initiatives and program aimed at mums of young children to encourage healthy eating and feeding habits.

We discovered beneficial relationships between the children's MDD and the rich home wealth index. Compared to women from households with the lowest wealth index, those from wealthy households had increased probabilities of seeking to give their children MDD. Other favorable research works from the Gambia [44], Burkina Faso [45], Rwanda [34], and Ethiopia [24]. The lack of resources in poor homes to offer a balanced diet for the family members may account for the link between a low wealth index and under adequate MDD in children. Such households frequently experience poor environmental conditions, a higher risk of disease exposure, and limited access to essential medical services, less ability to purchase household goods to their families as a whole might facilitate the inadequate MDD. Additionally, this study discovered that media exposure, access, and use—including reading newspapers, magazines, and other publications—were all positively related to attaining a sufficient MDD. This outcome was consistent with earlier research done in Ethiopia [24], Spain [46], and Rwanda [34]. This demonstrates the effectiveness of mass media exposure in modifying mothers' knowledge, attitudes, and health-seeking behaviors as well as their interactions with their families and the larger community.

The minimum dietary diversity of infants aged 6–23 months was similarly related to local government districts. Compared to mothers who had lived in Banjul, mothers who had lived in Kanifing, Janjanbureh, and Basse were 49%, 38%, and 51% less likely to provide their kids the necessary variety of meals. Although the author could not find other previous literatures to support this figure, we can have a solid evidence at hand to justify it according to the profiles of these local governmental areas. Evidence indicated that there are also regional differences in the distribution of health professionals. The Greater Banjul Area (GBA), which includes the Kanifing and Banjul City Councils, is home to the majority of health professionals in both the public and private sectors. Actually, of all the cadres, GBA has the highest percentage. The GBA contains more than half of the physician specialists, dentists, pharmacists, and laboratory workers. According to sector HR statistics, most (79%) of salaried professional health employees are working in the public sector, and majority of the urban residence found in Banjul, this population might have higher access of health-related practices [47, 48]. From this vantage point, most mums who reside in GBA may have a higher likelihood of receiving expert guidance concerning their children from these persons. Mothers that are knowledgeable and educated may be found here as well.

This study had a number of advantages. First of all, because the study was based on sizable datasets that were nationally representative, it has enough statistical power to generalize findings to Gambia children between the ages of 6 and 23 months. Second, to guarantee that the findings were representative at the national and regional levels, the estimates for the study were finished after the data had been weighted to account for probability sampling and nonresponse. Additionally, a suitable multilevel analytical technique was employed to study the determinants of changes in MDD over time. This will be the first national study in the Gambia and will provide insight for upcoming researchers and programmers. When analyzing the findings, it is important to consider the current study's several limitations. Because of the cross-sectional nature of the study, it is hard to determine whether the independent and dependent variables are causally related. Additionally, this study did not consider a number of unobserved variables, such as soil restrictions and altitude, which were not examined as part of the survey. The restrictions mentioned above should be taken into consideration when interpreting the survey's results. However, we think that our discoveries and suggestions will significantly advance knowledge about Gambian children's feeding practices.

## Conclusions

Although the MDD value may be marginally higher than certain countries' figures, the WHO dietary evaluation tool recommends that it is very low. The nation may require aggressive action to combat child malnutrition. After controlling confounders and other possible biases, mass media exposure, age of the child, wealth index and regions were the prominent factors that have been revealed a statistically significant value. The government of the Gambia may reap significant benefits if it supports local and national media, supports economically disadvantaged households, and collaborates with local states, community-based campaign implementers, and other stakeholders.

## Data Availability

Data for this analysis came from the DHS Program on Gambian 2019/20 DHS data files, which are open to the public. The DHS program repository has the datasets created and/or analyzed during the current investigation at www.dhsprogram.com.
